# Discovery of Nuclear-Encoded Genes for the Neurotoxin Saxitoxin in Dinoflagellates

**DOI:** 10.1371/journal.pone.0020096

**Published:** 2011-05-18

**Authors:** Anke Stüken, Russell J. S. Orr, Ralf Kellmann, Shauna A. Murray, Brett A. Neilan, Kjetill S. Jakobsen

**Affiliations:** 1 Microbial Evolution Research Group (MERG), Department of Biology, University of Oslo, Oslo, Norway; 2 Department of Molecular Biology, University of Bergen, Bergen, Norway; 3 School of Biotechnology and Biomolecular Sciences and Australian Centre for Astrobiology, University of New South Wales, Sydney, Australia; 4 Sydney Institute of Marine Sciences, Mosman, New South Wales, Australia; 5 Department of Biology, Centre for Ecological and Evolutionary Synthesis (CEES), University of Oslo, Oslo, Norway; East Carolina University, United States of America

## Abstract

Saxitoxin is a potent neurotoxin that occurs in aquatic environments worldwide. Ingestion of vector species can lead to paralytic shellfish poisoning, a severe human illness that may lead to paralysis and death. In freshwaters, the toxin is produced by prokaryotic cyanobacteria; in marine waters, it is associated with eukaryotic dinoflagellates. However, several studies suggest that saxitoxin is not produced by dinoflagellates themselves, but by co-cultured bacteria. Here, we show that genes required for saxitoxin synthesis are encoded in the nuclear genomes of dinoflagellates. We sequenced >1.2×10^6^ mRNA transcripts from the two saxitoxin-producing dinoflagellate strains *Alexandrium fundyense* CCMP1719 and *A. minutum* CCMP113 using high-throughput sequencing technology. In addition, we used in *silico* transcriptome analyses, RACE, qPCR and conventional PCR coupled with Sanger sequencing. These approaches successfully identified genes required for saxitoxin-synthesis in the two transcriptomes. We focused on *sxtA*, the unique starting gene of saxitoxin synthesis, and show that the dinoflagellate transcripts of *sxtA* have the same domain structure as the cyanobacterial *sxtA* genes. But, in contrast to the bacterial homologs, the dinoflagellate transcripts are monocistronic, have a higher GC content, occur in multiple copies, contain typical dinoflagellate spliced-leader sequences and eukaryotic polyA-tails. Further, we investigated 28 saxitoxin-producing and non-producing dinoflagellate strains from six different genera for the presence of genomic *sxtA* homologs. Our results show very good agreement between the presence of *sxtA* and saxitoxin-synthesis, except in three strains of *A. tamarense*, for which we amplified *sxtA*, but did not detect the toxin. Our work opens for possibilities to develop molecular tools to detect saxitoxin-producing dinoflagellates in the environment.

## Introduction

Saxitoxin and its derivatives (STX) are environmental neurotoxins, with significant economic, environmental and human health impacts. An estimated 2000 cases of human paralytic shellfish poisoning, with a mortality rate of 15%, occur globally each year [1]. The costs of monitoring and mitigation of STX have led to an annual economic loss from harmful plankton blooms calculated at US $895 million [2].

A striking feature of STX is that these compounds are synthesised by organisms from two kingdoms of life. They are produced by eukaryotic marine dinoflagellates and by prokaryotic freshwater cyanobacteria [3], [4]. The toxins appear to be synthesized by similar processes; precursor incorporation patterns and stereochemistry are identical in cyanobacteria and dinoflagellates [5].

The biosynthetic pathway and the genes responsible for STX-synthesis have been recently identified in the cyanobacterial species *Cylindrospermopsis raciborskii* [6], *Anabaena circinalis* [7], [8], *Aphanizomenon* sp. [7], *Raphidiopsis brookii* [9] and *Lyngbya wollei* [10]. Each cyanobacterial *sxt* gene cluster contains a set of core genes, common to all *sxt* clusters and a set of genes that are vary between different clusters [11], [12].

In contrast to cyanobacteria, the genetic basis for STX-production in dinoflagellates has remained elusive. Many studies have attempted to identify genes or enzymes involved in this pathway; through enzymatic characterisation [13], [14], PCR approaches [15], [16], [17], [18], *in silico* analyses of expressed sequence tag (EST) libraries [8], [19], or of other nucleotide sequences publicly available [20]. Despite these efforts, only one EST from the STX-producing *Alexandrium catenella* strain ACC07 has been identified as homologous to the N-terminal end of *sxtA* [8]. *SxtA* is the unique starting gene of STX-synthesis in cyanobacteria. It has four catalytic domains with predicted activities of a SAM-dependent methyltransferase (*sxtA1*), GCN5- related N-acetyltransferase (*sxtA2*), acyl carrier protein (*sxtA3*) and a class II aminotransferase (*sxtA4*) [6]. The origin of this unique enzyme may be chimeric: the domains *sxtA1-3* are most similar to extant proteobacterial sequences, whereas *sxtA4* may have a separate origin, possibly in actinobacteria [8].

Currently, it is unclear whether the synthesis of the same STX compounds, apparently via the same biosynthetic processes in bacteria and eukaryotes is a result of convergent evolution, horizontal gene transfer, or due to autonomous STX-production by bacteria associated with the dinoflagellate cell. The latter hypothesis has been investigated by a multitude of studies, but the results are conflicting. Some studies report an autonomous synthesis of STX by bacteria isolated from dinoflagellate cells (reviewed in [21]), whereas others show that axenic cultures of dinoflagellates may also produce STX [22]. In addition, methods used for measurements of bacterial STX lacked specificity, since compounds originally thought to be STX, have later been shown to be imposters [23], [24], [25], [26].

To clearly establish whether STX is produced by dinoflagellates it is necessary to identify the genes responsible for STX-production in STX-producing dinoflagellate cultures. The gene and transcript structure of bacteria and dinoflagellates are strikingly different. In dinoflagellates, genes may occur in multiple identical or non-identical copies (e.g. [27], [28]). The copy-number and sequence variation is reflected in their transcriptomes (e.g. [29], [30]). Dinoflagellate transcripts of nuclear encoded genes have polyA-tails and a unique dinoflagellate specific spliced-leader (SL) sequence [31], traits that have not been reported in bacteria. Spliced-leader sequences are small, non-coding RNAs that are trans-spliced onto the 5′end of mRNAs. In dinoflagellates, all nuclear-encoded genes appear to be trans-spliced with a conserved 22 base pair (bp) leader sequence, the dinoflagellate-SL [28], [31], [32]. This process converts polycistronic transcripts into translatable monocistronic mRNAs [33]. In contrast, bacterial transcripts may be polycistronic, such as the *sxt* gene cluster of *C. raciborskii* T3, where 24 genes are transcribed into five different mRNAs [34].

To identify *sxt* genes from two STX-producing *Alexandrium* cultures, we sequenced a large number of transcripts using high-throughput sequencing technology. In addition, we used *in silico* transcriptome analyses, rapid amplification of cDNA ends (RACE), qPCR and conventional PCR coupled with Sanger sequencing. These multiple approaches successfully identified genes required for STX-synthesis in dinoflagellates and show that these eukaryotes are able to produce STX autonomously.

## Methods

### Culturing and toxin measurements

Saxitoxin-producing and non-producing dinoflagellate cultures were obtained from various culture collections (Table 1). Cultures were maintained in GSe [35] or L1 media [36] at 16–20°C, under a 12/12 light cycle, and a photon irradiance of ∼100 micromoles of photons m^−2^ s^−1^. Toxicity of strains was determined using HPLC at the Norwegian Veterinary Institute, Oslo, Norway [37] or LCMS at the Cawthron Institute, Nelson, New Zealand. The detection limit of the HPLC method ranged from about 0.07 µg STXeq/100 g for C1 and C3 to 4.1 µg STXeq/100 g for GTX1. The detection limit for the LCMS method ranged from about 0.1 pg/cell for NEO and STX to 0.5 pg/cell for C1 and C2.

**Table 1:** List of dinoflagellate strains used in this study, their production of STX and whether *sxtA1* and *sxtA4* fragments were amplified from their genomic DNA.

ORDER *Genus Species*	Strain	STX	PCR sxtA1	PCR sxtA4
GONYAULACALES				
*Alexandrium affine*	CCMP112	n. d.	n. d.	n. d.
*Alexandrium affine*	AABB01/01	n. d.	n. d.	n. d.
*Alexandrium affine*	AABB01/02	n. d.	n. d.	n. d.
*Alexandrium andersonii*	CCMP1597	n. d.	n. d.	n. d.
*Alexandrium andersonii*	CCMP2222	n. d.	n. d.	n. d.
*Alexandrium catenella*	ACCC01	yes	yes	yes
*Alexandrium catenella*	ACSH02	yes	yes	yes
*Alexandrium catenella*	ACTRA02	yes	yes	yes
*Alexandrium catenella*	CCMP1493	yes	yes	yes
*Alexandrium fundyense*	CCMP1719	yes	yes	yes
*Alexandrium fundyense*	CCMP1979	yes	yes	yes
*Alexandrium minutum*	CCMP1888	yes	yes	yes
*Alexandrium minutum*	CCMP113	yes	yes	yes
*Alexandrium minutum*	ALSP01	yes	yes	yes
*Alexandrium minutum*	ALSP02	yes	yes	yes
*Alexandrium minutum*	AMD16	yes	yes	yes
*Alexandrium tamarense*	CCMP1771	n. d.	yes	yes
*Alexandrium tamarense*	ATBB01	n. d.	yes	yes
*Alexandrium tamarense*	ATEB01	n. d.	yes	yes
*Alexandrium tamarense*	ATCJ33	n. d.	yes	yes
*Alexandrium tamarense*	ATNWB01	yes	yes	yes
*Gambierdiscus australes*	CAWD148	*	n. d.	n. d.
*Ostreopsis ovata*	CAWD174	*	n. d.	n. d.
*Ostreopsis siamensis*	CAWD96	*	n. d.	n. d.
GYMNODINIALES				
*Amphidinium massarti*	CS-259	*	n. d.	n. d.
*Gymnodinium catenatum*	GCTRA01	yes	yes	yes
*Gymnodinium catenatum*	CS-395	yes	yes	yes
PROROCENTRALES				
*Prorocentrum lima*	CS-869	*	n. d.	n. d.

n.d. not detected,

*species never reported to synthesize STX.

### RNA and DNA extraction

To isolate total RNA for the 454-library construction (see below), cultures of *Alexandrium fundyense* Balech CCMP1719 and *Alexandrium minutum* Halim CCMP113 were harvested in exponential phase through centrifugation (1 min, 1000× *g*, 12°C). Cells were washed with PBS, exposed to bead-beating on dry ice with the Fast Prep bead-beater from Medinor (20 s, speed 4) using 1.4 mm beads (Medinor) and total RNA was extracted with the ChargeSwitch® Total RNA Cell kit (Invitrogen) according to the manufacturers' protocol.

For RACE analyses, polyA-enriched mRNA was isolated using the Dynabeads DIRECT kit (Invitrogen). Cells were harvested by centrifugation (2 min, 4°C, 16000× *g*), were washed twice with PBS, the lysis/binding buffer was added, and this was homogenised using the bead-beater (20 s, step 4). After centrifugation (1 min, 4°C, 16000× *g*), the clear homogenate was transferred to the Dynabeads mix and the mRNA isolated according to protocol. Finally, mRNA was treated with TURBO™ DNase (Ambion) according to the protocol supplied.

Genomic DNA was isolated from all dinoflagellate strains listed in Table 1 by either using the Genomic DNA plant ChargeSwitch® kit (Invitrogen) according to the manufacturer's protocol, or by the CTAB method [38].

Quality and quantity of RNA and DNA were determined using a Nanodrop spectrophotometer (ThermoScientific), by amplifying control dinoflagellate genes (cytochrome b, actin) and/or by visualizing them on an ethidium bromide stained agarose gel.

### cDNA library construction, 454 sequencing, assembly and analyses

Normalized polyA-enriched complementary DNA (cDNA) libraries with 454 adapters attached at each end were constructed commercially by Vertis Biotechnologie AG (http://www.vertis-biotech.com/). Half a plate each of *A.fundyense* CCMP 1719 and *A. minutum* CCMP113 libraries were sequenced using Roche 454 sequencing TITAN technology at the Norwegian High-Throughput Sequencing Centre (http://www.sequencing.uio.no/). Only 454 reads that possessed at least one cDNA adaptor were considered further. Adaptors and, where present, full and partial dinoflagellate spliced-leader (SL) sequences were removed prior to assembly using an in-house PERL script which is now integrated in the bioinformatic tool CLOTU [39]. Reads were assembled using the software program Mira v3.0.5 [40] with the main switches ‘denovo’, ‘est’, ‘accurate’ and ‘454’.

To identify putative *sxt* gene sequences within the two 454 libraries, custom BLAST searches were performed at the freely available online data portal ‘Bioportal’ (www.bioportal.no). Two strategies were used: the cyanobacterial *sxt* genes were queried either against the assembled *Alexandrium* datasets or the unassembled 454 read datasets. All hits with an e-value<0.1 were extracted and the sequence with the lowest e-value for each gene was blasted against the non-redundant protein database at NCBI.

For *sxtA*, all retrieved sequences were re-assembled in the software program CLC Bio Main Workbench, using a minimum overlap of 10 bp and low or high alignment stringency. Resulting contig sequences were blasted against the non-redundant and EST databases at NCBI using algorithms blastn, blastx and tblastx. The structure of *sxtA* transcripts was determined by aligning their translated sequence to *sxtA* from cyanobacteria, as well as by conserved domains searches (http://www.ncbi.nlm.nih.gov/Structure/cdd/wrpsb.cgi). Catalytic and substrate-binding residues of *sxtA* from cyanobacteria have been previously determined [6], [41]. The transcripts were searched for the presence possible signal peptides and corresponding cleavage sites using the neural networks and hidden Markov models implemented in SignalP 3.0 ([42] http://www.cbs.dtu.dk/services/SignalP/) and the 3-layer approach of Signal-3L ([43] http://www.csbio.sjtu.edu.cn/bioinf/Signal-3L/). Transmembrane helices were explored using TMHMM server 2.0 (http://www.cbs.dtu.dk/services/TMHMM/) and hydrophobicy profiles with Kyte-Doolittle plots [44].

### RACE analyses

Primers were designed in conserved regions of the contigs with high similarity to *sxtA* using Primer3 software (http://frodo.wi.mit.edu/primer3/; Table 2). First-strand cDNA was synthesized with ∼95 ng polyA-enriched mRNA using the adaptor primer AP according to the manufacturer's instructions for transcripts with high GC content (3′RACE System, Invitrogen). Following RNase H treatment, the RACE product was 1∶10 diluted and used as template for PCR. To amplify the 5′end of the transcript, three different protocols were used. First, the method of Zhang *et al.* [31] was used with slight modifications: the 3′RACE library described above was amplified with the primers AUAP (adapter primer supplied with the kit) and dinoSL [31] to enrich for full transcripts (PCR program: 94°C - 60 s; 30×(94°C - 30 s, 68°C - 5 min); 68°C - 10 min; 8°C hold; PCR chemistry see below). The PCR product was 1∶10 diluted and used as template in nested PCRs, which were amplified using the dinoSL primer as forward and several different internal reverse primers (Table 2). Further, we used the two kits 5′RACE System (Invitrogen) and the GeneRacer kit (Invitrogen), using the provided 5′Adapter primers and several different internal reverse primers (Table 2). All products were cloned and sequenced as described below.

**Table 2:** Primers used in PCR and sequencing.

Name	Sequence 5′ - 3′	Orientation	Description
sxt001	TGCAGCGMTGCTACTCCTACTAC	Forward	binds within sxtA1, designed on 454 reads
sxt002	GGTCGTGGTCYAGGAAGGAG	Reverse	binds within sxtA1, designed on 454 reads
sxt007	ATGCTCAACATGGGAGTCATCC	Forward	binds within sxtA4, designed on 454 reads
sxt008	GGGTCCAGTAGATGTTGACGATG	Reverse	binds within sxtA4, designed on 454 reads
sxt013	GTAGTAGGAGTAGCKACGCTGCA	Reverse	reverse complement of sxt001
sxt014	CTCCTTCCTRGACCACGACC	Forward	reverse complement of sxt002
sxt015	GGATGACTCCCATGTTGAGCAT	Reverse	reverse complement of sxt007
sxt016	CATCGTCAACATCTACTGGACCC	Forward	reverse complement of sxt008
sxt019	GGCAAGTATCTCCGCAGGCTTAC	Reverse	binds within sxtA1, upstream of sxt002
sxt020	CGTGGAGGAGCATGTTGACAGAATC	Forward	binds within sxtA1, downstream of sxt001
sxt026	ACTCGACAGGCCGGCAGTACAGAT	Reverse	binds with sxtA4, upstream of sxt008
sxt040	TGAGCAGGCACGCAGTCC	Forward	binds within sxtA1 on the long transcript
TopoF	GGCTCGTATGTTGTGTGGAATTGTG	Forward	binds within pCR®2.1-TOPO® vector
TopoR	AGTCACGACGTTGTAAAACGACGG	Reverse	binds within pCR®2.1-TOPO® vector

### PCR and sequencing

All PCR reactions were carried out in 25 µl volumes containing template, 1 unit 10× BD Advantage 2 PCR buffer (BD Biosciences), 0.2 mM dNTPs, 0.5 µM of each forward and reverse primer (Table 2), DMSO (10% final concentration) and 0.25 units 50× BD Advantage 2 Polymerase Mix (BD Biosciences). If not stated otherwise, PCRs were amplified as follows: 94°C - 2.5 min; 5×(94°C - 30 s; 68°C - variable); 5×(94°C - 30 s; 66°C - 30 s; 68°C - variable); 25×(94°C - 30 s; 64°C - 30 s; 68°C - variable); 68°C - 10 min; 8°C – hold. PCR products were visualized on 1% ethidium bromide stained agarose gels, cut out and cleaned with the Wizard® SV Gel and PCR Clean-up System (Promega) and cloned with the TOPO TA® cloning kit according to the manufacturer's instructions (Invitrogen; pCR®2.1-TOPO® vector; One Shot® Mach1™ T1 Phage-Resistant Chemically Competent E. coli cells). Individual colonies were directly added to 25 µl PCR reactions containing 1 unit 10× standard PCR buffer (Qiagen), 0.4 µM primer TopoF and TopoR (Table 2), 0.2 mM dNTPs, and 1 unit HotStarTaq (Qiagen). Cycling conditions were 95°C - 15 min, 30×(94°C - 30 s; 60°C - 30 s; 72°C - 90 s), 72°C - 5 min, 8°C - hold. PCR products were diluted and Sanger sequenced directly from both sides using the primers M13F and M13R supplied with the cloning kit.

### *SxtA1* and *sxtA4* genomic amplification

All dinoflagellate strains (Table 1) were tested for the presence of putative *sxtA1* and *sxtA4* genes. PCRs were run using gDNA according to the protocol described above. The *sxtA1* fragment was amplified with primers sxt001 & sxt002 (∼550 bp) and the *sxtA4* fragment with the primers sxt007 & sxt008 (∼750 bp) (Table 2).

### Phylogenetic analyses

Dinoflagellate nucleotide sequences were aligned manually using MacClade v4.07 [45] considering the coding sequence in the correct reading frame before being translated to the corresponding amino-acid sequence. The dinoflagellate amino acid sequences were subsequently aligned, using MAFFTv6 L-INS-I model [46] to the orthologous *sxt* sequences for cyanobacteria, in addition to a selection of closely related NCBI nr Blastp hits, constituting the outgroup. Resulting alignments were checked manually and poorly aligned positions excluded using MacClade v4.07 [45].

ProtTest v2.4 [47] determined WAG as the optimal evolutionary model for all inferred alignments. Maximum Likelihood (ML) analyses were performed with RAxML-VI-HPCv7.2.6, PROTCATWAG model with 25 rate categories [48]. The most likely topology was established from 100 separate searches and bootstrap analyses were performed with 100 pseudo-replicates. Bayesian inferences were performed using Phylobayes v3.2e [49], [50] under the same substitution model with a free number of mixing categories and a discrete across site variation under 4 categories. Trees were inferred when the largest maximum difference between the bipartitions (chains) was <0.1. All model estimation and phylogenetic analyses were done on the freely available ‘Bioportal’ (http://www.bioportal.uio.no/).

### Copy number determination

Triplicate 200 ml batch cultures of *Alexandrium catenella* strain ACSH02 were grown as previously described, and abundance was counted every three days using a Sedgewick-Rafter chamber and inverted light microscope (Leica Microsystems). Ten ml samples for gDNA extraction were taken in early exponential, late exponential and stationary phase.

Primers suitable for qPCR were designed based on conserved regions in an alignment of *A. fundyense* and *A. minutum* 454 reads covering the *sxtA4* region using Primer 3 software amplifying a 161 bp product. qPCR cycles were carried out on a Rotor Gene 3000 (Corbett Life Science) using SYBR Green PCR Master Mix (Invitrogen). qPCR assays were performed in a final volume of 25 µl volume consisting of 12.5 µl SYBR Green PCR master mix, 1 µl of template DNA, 1 µl of each primer pair, 1 µl of BSA and 8.5 µl of MilliQ water. qPCR assays were performed in triplicate with the following protocol: 95°C for 10 s, and 35 cycles of 95°C for 15 s and 60°C for 30 s. Melting curve analysis was performed at the end of each program to confirm amplification specificity, and select PCR products were sequenced. The standard curve was constructed from a 10-fold dilution series of a known concentration of fresh PCR product, ranging from 2–2×10^−5^ ng. The molecules of PCR product were determined: (A×6.022×10^23^)×(660×B)^−1^ with A: concentration of PCR product, 6.022×10^23^: Avogadro's number, 660: average molecular weight per base pair and B: length of PCR product. The number of molecules in the unknown samples were determined and divided by the known number of cells in the qPCR template to obtain copy number per cell. The detection limit was around 5000 copies of the gene sequence (i.e. ∼20–30 cells per assay, each with ∼200 copies of the sequence). However, the analyses were run with 10–100-fold this number of cells, and thus not run at or close to the detection limit.

## Results

### Identification of *sxt* sequences in the transcriptome of *A. minutum* and *A. fundyense*

454 sequencing resulted in 589,410 raw reads for *A. minutum* and 701,870 raw reads for *A. fundyense* (SRA028427.1: samples SRS151150.1 and SRS151148.1, respectively). After quality control, the reads were assembled into 44,697 contigs and 539 singletons for *A. minutum* and 51,861 contigs and 163 singletons for *A. fundyense*. The contig lengths and GC contents were similar for both libraries: the mean sequence lengths (± SD) of 669 bp (±360) and 678 bp (±361) and a GC content of 59% and 58% were calculated for *A. minutum* and *A. fundyense*, respectively.

Searching the unassembled 454 cDNA library reads with the cyanobacterial *sxtA* gene resulted in 94 hits for *A. fundyense* and 88 hits for *A. minutum*, respectively. The same search on the assembled datasets returned 10 contigs from the *A. fundyense* and 9 from the *A. minutum* library. After pooling of all sequences and re-assembly, two contigs showed a high similarity to *sxtA* from cyanobacteria: one to the domain *sxtA1* (contig length = 1450 bp, GC = 60.1%, bit score = 213, e-value = 5e^−61^) and the other to *sxtA4* (contig length = 1059 bp, GC = 65%, bit score = 195, e-value = 1e^−47^). Both contigs contained sequences from both *Alexandrium* libraries, but neither contained a full ORF, a dinoflagellate spliced leader sequence or a polyA-tail. The two contigs were used to design *sxtA1* and *sxtA4* primers for genomic amplification, RACE analyses and sequencing.

The results of the *in silico* search for the remaining core *sxt* genes are summarized in Table 3. Apart from *sxtA*, contigs with a good alignment score (bit score >55) and a highly significant e-value (<e^−20^) were recovered for the amidinotransferase gene *sxtG* in both libraries. Re-blasting the contigs with the lowest e-values against the NCBI nr protein database showed that the most similar gene was an actinobacterial glycine aminotransferase, while the similarity to *sxtG* from cyanobacteria was less but still highly significant (Table 3). For the core biosynthesis genes *sxtB*, *sxtF/M*, *sxtH/T*, *sxtI*, *sxtR* and *sxtU*, contigs with an e-value≤0.1 were recovered from both *Alexandrium* libraries, while *sxtS* only had a hit in the *A. minutum* library (Table 3). No matches were recovered for *sxtC*, *sxtD* and *sxtE* in either of the libraries. *SxtC* and *sxtE* are unknown proteins and *sxtD* is a sterol desaturase-like protein [6]. It is possible that dinoflagellate proteins with no similarity to the cyanobacterial genes carry out their function. Alternatively, these genes were not present in our dataset. While our dataset is comprehensive, it is not complete. For example, some regions of the *sxtA* transcripts were also not recovered in the 454 dataset, but only obtained through RACE analyses (see above). Re-blasting against NCBI nr protein database retrieved hits to proteins for *sxtB* (*A. fundyense* only), *sxtF/M*, sxtH/T, *sxtI*, and *sxtU* that are similar to those encoded in the corresponding cyanobacterial *sxt* genes. The actual sequence similarity was less conserved and no significant hits between the *Alexandrium* contigs and the cyanobacterial *sxt* genes were observed.

**Table 3:** Blast analyses of the core *sxt* genes from *C. raciborskii* T3 against the assembled *A. fundyense* and *A. minutum* 454 libraries.

454 library	Number of contigs	Top score/E-value	Uppermost blastX hit of top contig against NCBI nr-database	Accession	Taxonomy	Uppermost blastX score/E-value	Top *sxt* hit score/E-value
***sxtA***							
*A. fundyense*	10	105/2e^−51^	polyketide synthase [*Myxococcus xanthus* DK 1622]	YP_63211	Bacteria; Proteobacteria	183/5e^−44^	182/7e^−44^
*A. minutum*	9	108/3e^−61^	SxtA [*Lyngbya wollei*]	ACG63826	Bacteria; Cyanobacteria	236/2e^−65^	236/2e^−65^
***sxtB***							
*A. fundyense*	1	46/7e^−11^	cytidine deaminase [*Plesiocystis pacifica* SIR-1]	ZP_01910517	Bacteria; Proteobacteria	91/9e^−27^	67/1e^−11^
*A. minutum*	1	35/0.094	none				
***sxtF/sxtM***							
*A. fundyense*	4	51/4e^−06^	putative efflux protein, MATE [*Polysphondylium pallidum* PN500]	EFA81712	Eukaryota; Amoebozoa	136/2e^−30^	62/5e^−08^
*A. minutum*	1	34/0.01	putative efflux protein, MATE [*Arabidopsis lyrata subsp. lyrata*]	XP_002873960	Eukaryota; Viridiplantae	78/8e^−23^	none
***sxtG***							
*A. fundyense*	9	57/2e^−27^	glycine amidinotransferase [*Amycolatopsis mediterranei* U32]	YP_003768377	Bacteria; Actinobacteria	163/3e^−38^	140/2e^−31^
*A. minutum*	7	55/2e^−25^	glycine amidinotransferase [*Amycolatopsis mediterranei* U32]	YP_003768377	Bacteria; Actinobacteria	143/2e^−32^	117/1e^−24^
***sxtH/sxtT***							
*A. fundyense*	7	43/2e^−12^	Rieske (2Fe-2S) region [*Anabaena variabilis* ATCC 29413]	YP_321575	Bacteria; Cyanobacteria	197/6e^−86^	80/1e^−12^
*A. minutum*	6	41/5e^−06^	Rieske (2Fe-2S) region [*Anabaena variabilis* ATCC 29413]	YP_321575	Bacteria; Cyanobacteria	119/5e^−38^	60/2e^−07^
***sxtI***							
*A. fundyense*	3	68/1e^−13^	Carbamoyltransferase [*Nocardiopsis dassonvillei* DSM 43111]	YP_003679504	Bacteria; Actinobacteria	131/9e^−29^	89/9e^−16^
*A. minutum*	1	67/1e^−13^	carbamoyl transferase [*Streptomyces griseoflavus* Tu4000]	ZP_05536710	Bacteria; Actinobacteria	132/6e^−29^	91/1e^−16^
***sxtR***							
*A. fundyense*	3	36/0.063	atp-citrate synthase [*Ectocarpus siliculosus*]	CBJ30109	Eukaryota; Stramenopiles	349/8e^−96^	none
*A. minutum*	1	38/0.015	atp-citrate synthase [*Ectocarpus siliculosus*]	CBJ30109	Eukaryota; Stramenopiles	516/1e^−144^	none
***sxtS***							
*A. minutum*	1	36/0.05	hypothetical protein [*Perkinsus marinus* ATCC 50983]	XP_002767298	Eukaryota; Alveolata	91/4e^−34^	none
***sxtU***							
*A. fundyense*	33	83/2e^−16^	predicted protein [*Chlamydomonas reinhardtii*]	XP_001689640	Eukaryota; Viridiplantae	214/4e^−54^	107/8e^−22^
*A. minutum*	27	84/2e^−16^	hypothetical protein [*Schizophyllum commune* H4-8]	XP_003034688	Eukaryota; Fungi	116/1e^−24^	797/2e^−13^

Given are: the number of contigs with an E-value≤0.1 present in each library; the top blastX hit, its accession number, taxonomy, score and E-value when the top contig is blasted against the non-redundant protein database of NCBI, as well as the closest hit to *sxt* genes from cyanobacteria from the same analysis.

### Transcript structure of *sxtA in A. fundyense*

**Figure 1:**
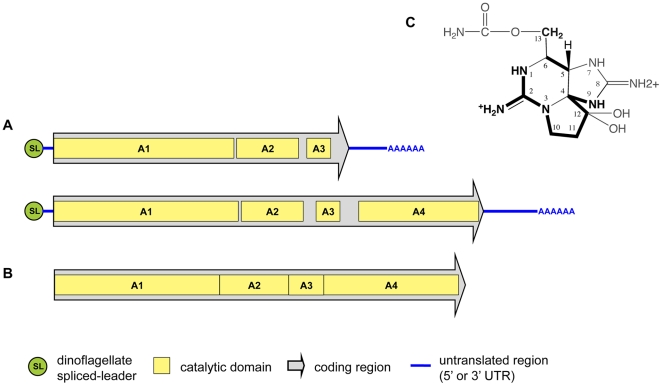
The structure of *sxtA* in dinoflagellates and cyanobacteria. a) Transcript structure of *sxtA* transcripts in *A. fundyense* CCMP1719. b) Genomic *sxtA* structure of *C. raciborskii* T3. c) Structure of STX with bonds and molecules introduced by *sxtA* marked in bold.

The RACE experiments resulted in two different *sxtA* - like transcript families. Both had dinoflagellate spliced-leader sequences at the 5′end and polyA-tails at the 3′end, but they differed in sequence, length, and in the number of *sxt* domains they encode. The shorter transcripts encode the domains *sxtA1*, *sxtA2* and *sxtA3*, while the longer transcripts encodes all four *sxtA* domains, which are also encoded by the cyanobacterial *sxtA* gene (Fig. 1).

The consensus sequence of the shorter transcripts was 3136 bp excluding polyA-tail. Eight clones with SL-leader were sequenced, and three different 5′UTRs were uncovered. The sequences were almost identical; however, one clone had a 15 bp and another had a 19 bp insert exactly following the SL-sequence. The two sequence inserts were, apart from the length, identical. The nine 3′UTR that were sequenced were almost identical and the polyA-tail started at the same position in each clone. The domain structure of this shorter *sxtA* transcript was as follows: Amino acid residues 1-27 encode a signal peptide. Residues 28-531 correspond to *sxtA1*, which contains three conserved motifs (I: VDTGCGDGSL, II: VDASRTLHVR, III: LEVSFGLCVL). Residues 535-729 correspond to *sxtA2* with the catalytic domains 557-W, 648-T, 663-H, 711-R; while *sxtA3*, the final domain of the short transcript, corresponds to residues 750-822 with the phosphopantetheinyl attachment site 783-DSL-785.

The consensus sequence of the longer *sxtA* transcript was 4613 bp (majority rule, longest 3′UTR, without polyA-tail, Fig. 1). Five clones with SL-sequences were characterized. One of those had a slightly divergent SL-sequence with an A at position 15 instead of the usual G. All 5′UTRs were 97 bp long (excluding SL sequence) and almost identical in sequence. Each of the four 3′clones sequenced had a different length (342, 407, 446 and 492 bp). The domain structure of the longer *sxtA* transcript was as follows: Amino acid residues 1-25 encode a signal peptide. Residues 26-530 correspond to domain *sxtA1* with the three conserved motifs: I: VVDTGCGDG, II: VDPSRSLHV and III: LQGSFGLCML; residues 535-724 correspond to domain *sxtA2*, with the catalytic residues 556-W, 661-T, 693-H, 708-R; *sxtA3* corresponds to the residues 763-539 where 799-DSL-801 is the phosphopantetheinyl attachment site; finally, domain *sxtA4* corresponds to residues 894-1272.

**Figure 2:**
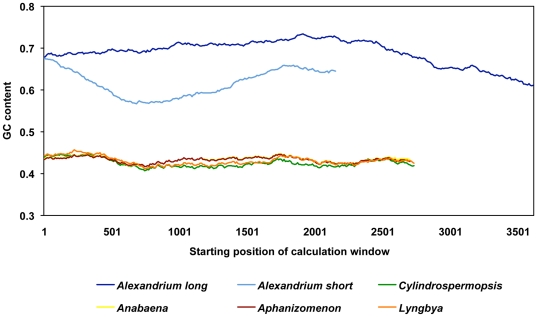
GC content of *A. fundyense sxtA* transcripts and of cyanobacterial *sxtA* genes. GC content was calculated every 10 bp with a window size of 1000 bp.

The GC content of the two *Alexandrium sxtA* transcripts was consistently higher than the cyanobacteria *sxtA* genes (Fig. 2). The GC contents were 69% (long transcript), 62% (short transcript) and 43% (all cyanobacteria *sxtA* genes).

All algorithms predicted the presence of signal peptides (SP) and corresponding cleavage sites for both transcripts (Supporting Information S3). However, transmembrane helices that may indicate class I transit peptides in dinoflagellates [51] were not predicted. Neither of the transcripts matched the criteria for class II and class III transit peptides [51].

The Genbank accession numbers are JF343238 for the short and JF343239 for the long *sxtA* transcripts (majority rule consensus sequences), and JF343357–JF343432 for the remaining cloned RACE sequences of *A. fundyense* CCMP 1719.

### Phylogeny of dinoflagellate *sxtA1* and *sxtA4* sequences

The *sxtA1* and *sxtA4* primers designed in this study (Table 2) amplified single bands of ∼550 bp (*sxtA1*) and ∼750 bp (*sxtA4*) length in 18 *Alexandrium* strains comprising five species and two *Gymnodinium catenatum* strains, which had a range of toxicities (Table 1). No *sxtA1* or *sxtA4* PCR products were amplified for five non-STX-producing *Alexandrium affine* and *Alexandrium andersonii* strains, nor for non-STX-producing dinoflagellate strains of the genera *Gambierdicus*, *Ostreopsis*, *Prorocentrum*, *Amphidinium* (Table 1). These PCR-based results are generally in agreement with the toxin measurements. However, *sxtA1* and *sxtA4* fragments were amplified from the genomic DNA of four *A. tamarense* strains (ATCJ33, ATEB01, CCMP1771, ATBB01) in which no STX were detected (Table 1).

**Figure 3:**
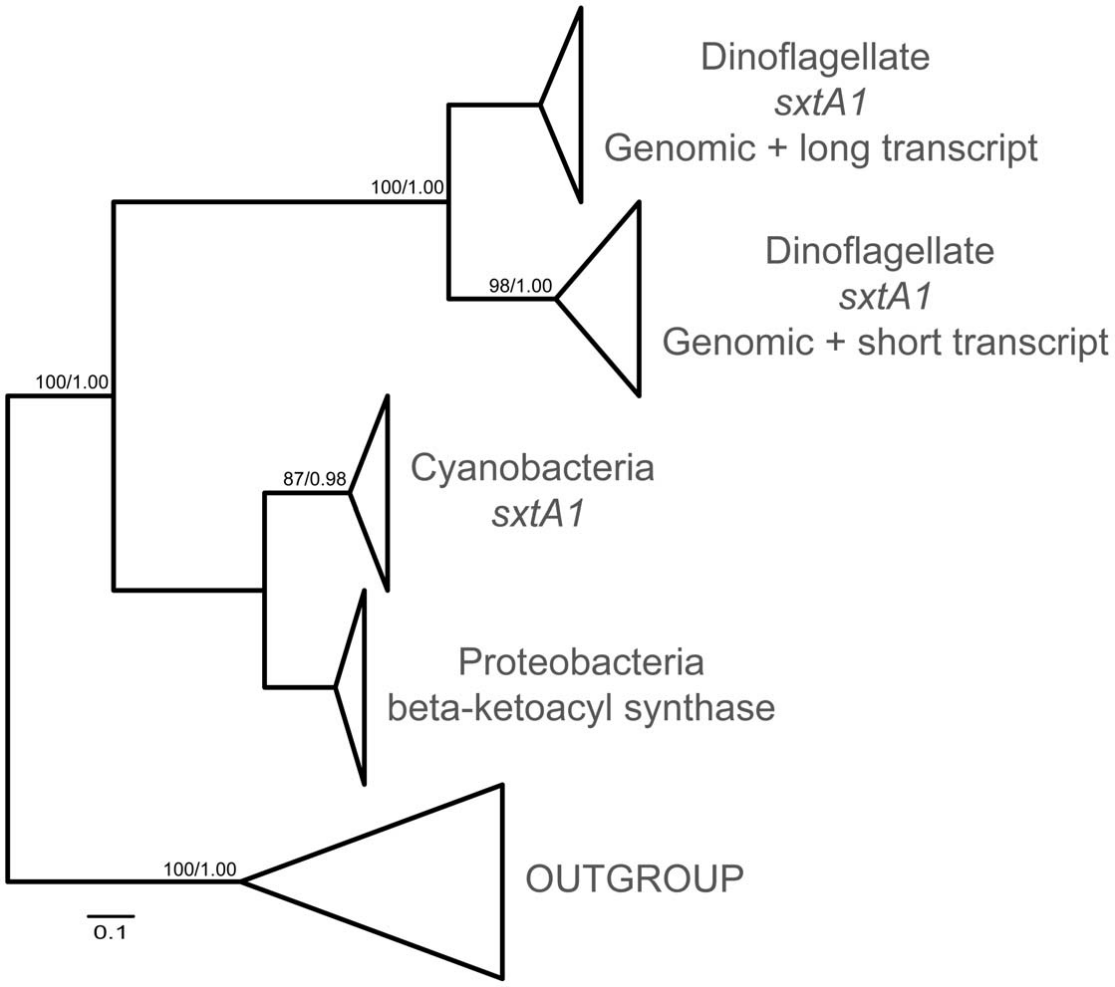
S*xtA1* phylogenetic tree. Schematic representation, drawn to scale (for full tree see Supporting Information S1). Maximum likelihood topology is shown. Numbers on nodes represent bootstrap values of maximum likelihood and Bayesian analyses, respectively.

The phylogenetic analyses of *sxtA1* (Fig. 3; Supporting Information S1) show that all *sxtA1* sequences formed one fully supported cluster, divided into two sub-clusters. Some clones of the same strain were identical, however, slightly different clones were observed for most strains (Supporting Information S1). These different clones were distributed throughout the phylogeny, generally without species- or strain-related patterns. Only sequences from *G. catenatum* formed a tight branch within one of the sub-clusters. The closest relatives to the dinoflagellate cluster were the cyanobacterial *sxtA* genes and proteobacterial polyketide synthases (Fig. 3; Supporting Information S1).

**Figure 4:**
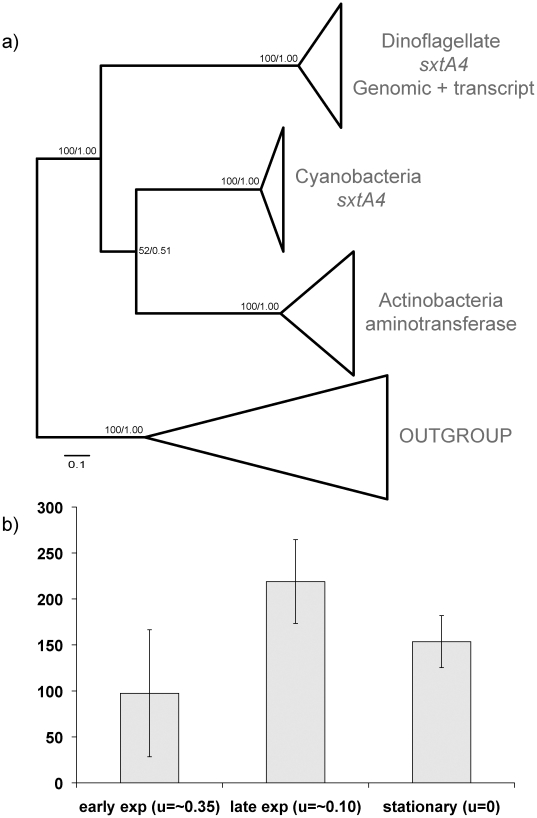
S*xtA4* phylogenetic tree and genomic copy number. a) Schematic representation of phylogenetic tree, drawn to scale (for full tree see Supporting Information S2). Maximum likelihood topology is shown. Numbers on nodes represent bootstrap values of maximum likelihood and Bayesian analyses, respectively. b) Genomic copy number of *sxtA4* in A. *catenella* ACSH02 at three different time-points during the growth cycle.

All *sxtA4* sequences formed one well-supported cluster, with clones from the same strain distributed throughout (Fig. 4; Supporting Information S2). The cyanobacterial *sxtA* genes and actinobacterial aminotransferases formed the closest sister clades.

The Genbank accession numbers for the genomic *sxtA1* and *sxtA4* fragments are JF343240–JF343356.

### Copy number and polymorphisms of *sxtA4*

Between 100–240 genomic copies of *sxtA4* in *A. catenella* were found in triplicate batch cultures of ACSH02 collected at three time points with different growth rates, based on the qPCR assay (Fig. 4b).

Analysis of a 987 bp contig, which covered the *sxtA4* domain and was based on *A. fundyense* 454 reads revealed at least 20 single nucleotide polymorphisms (SNPs), 15 of which were silent. SNPs were defined as a base pair change that occurred in at least two of the reads. Homopolymer stretches and indels were ignored.

## Discussion

### *Sxt* genes are encoded in dinoflagellate genomes

Until recently, the unusually large (1.5–200 pg DNA cell-1; [52]) and highly divergent genomes of dinoflagellates have hindered attempts to determine the genetic basis of their toxin production. Recent estimates predict that dinoflagellate genomes contain between 38,000 and almost 90,000 protein-encoding genes [53], which correspond to 1.5–4.5 the number of genes encoded in the human genome [54]. Advances in sequencing technology have made it possible to efficiently investigate the complex transcriptome of dinoflagellates. The results of sequencing >1.2 million ESTs in this study demonstrate that close homologues of the genes involved in STX biosynthesis in cyanobacteria are also present in STX-producing dinoflagellates (Table 3). To further confirm their dinoflagellate origins, we investigated *sxtA*, the unique starting gene of the biosynthesis pathway [6]. The transcriptome of *A. fundyense* CCMP 1719 contained two different transcript families that had the same domain architecture as *sxtA* in cyanobacteria. The two transcript families varied in length, sequence, and the number of catalytic domains they encode. The longer transcripts contained all four domains present in the known cyanobacterial *sxtA* genes, however, the shorter transcripts lacked the terminal aminotransferase domain (Fig. 1). In contrast to bacterial transcripts, both transcript families possessed eukaryotic polyA-tails at the 3′end and dinoflagellate spliced-leader sequences at the 5′end. Thus, our results clearly show that at least *sxtA*, and possibly other *sxt* genes, are encoded in the nuclear genome of dinoflagellates and that STX-synthesis in dinoflagellates does not originate from co-cultured bacteria. As has been proposed, these bacteria may still, however, play an important role in modulating STX biosynthesis in dinoflagellates [22], [55].

The signal peptides identified in both transcripts indicate a specific targeting of both Sxt products. Many genes in the nuclear genomes of dinoflagellates are plastid-derived and their products targeted to the plastid (e.g. [51]). These proteins are translated in the cytosol and then transported to the plastid through the plastid membranes. In peridinin-containing dinoflagellates like *Alexandrium*, this process requires the presence of signal and transfer peptide motifs [51]. Both *sxtA* transcripts are predicted to contain signal peptides, but transfer-peptide structures were not identified. Thus, it seems that both *sxtA* proteins are targeted out of the cytosol, but the region of target need to be experimentally investigated.

The dinoflagellate *sxtA* transcripts did not only differ from the cyanobacterial counterparts by the presence of signal peptides, SL sequences and polyA-tails, but also in their GC content. The *A. fundyense* ESTs had a considerably higher GC content (Fig. 2). Transcribed genes from *Alexandrium* species have been reported to have an average GC content >56% [56], [57], [58], [59], while filamentous cyanobacteria, such as the STX-producing genera *Cylindrospermopsis*, *Anabaena*, *Aphanizomenon* and *Lyngbya*, have a genomic GC content around 40% [9], [60], [61]. This indicates that the GC content of *sxtA* has diverged significantly from the progenitor *stxA* possessing ancestor, in line with the rest of the genome in these microorganisms.

Recent analyses of the codon usage patterns of the STX-producing *A. tamarense* strain CCMP 1598 suggest that mutational bias, translational selection, hydropathy and aromaticity influence the selection of codon use in this species, however, codon usage also differs between high and low level expressed genes [59]. The involvement of the two different *sxtA* transcripts and their role in STX-synthesis is presently unclear, but the differences in GC content (Fig. 2) indicate that they are under different selection pressures.

### The non-identical copies of *sxtA*: variation at the genome and transcriptome level

One typical feature of dinoflagellate genomes is that genes may occur in multiple copies, which may or may not be identical [27], [28]. This is possibly related to highly unusual genetic mechanisms such as the recycling of processed cDNAs [62]. It appears that *sxtA* also occurs in multiple copies within dinoflagellate genomes. We estimated that 100–240 copies of the *sxtA4* domain were present in the genomic DNA of *A. catenella* ACSH02 (temperate Asian ribotype). The copy number differences detected throughout the cell cycle are likely related to the growth rate of the batch culture and the proportion of cells in various cell cycle phases. All genomic *sxtA4* sequences from 15 different *Alexandrium* and one *G. catenatum* strains formed one well-supported phylogenetic cluster, with several slightly different clone sequences of the same strain distributed throughout the tree. *SxtA1* was also found to occur in multiple, non-identical copies in all strains analysed (Supporting Information S1). Further, the separation of the dinoflagellate *sxtA1* cluster into two sub-clades indicates that *sxtA1* may be encoded by two separate gene classes, at least in some strains.

The genomic variation of *sxtA* is also present in the *Alexandrium* transcriptomes. Adding the transcriptome data to the *sxtA1* tree showed that the upper clade corresponds to the longer *sxtA* transcripts, whereas the lower clade corresponds to the shorter transcripts (Fig. 3, Supporting Information S1). Analyses at the nucleotide level of the *sxtA4* region in the transcriptome of *A. fundyense* revealed many of SNP sites, two-thirds of which were silent. This is in line with results of other EST studies of dinoflagellate species, showing that gene families can comprise members with similar but non-identical sequences [28], [56]. Results from previous studies also indicate that much of the variation observed at the nucleotide level does not translate into variation in peptide structure [63].

### Correlation between *sxtA1*, *sxtA4* and saxitoxin production

The putative *sxtA1* and *sxtA4* genomic sequences identified during this study were present in all STX-producing dinoflagellate strains analysed, including two *G. catenatum* and 14 *Alexandrium* strains of the species *A. catenella*, *A. minutum*, *A. fundyense* and *A. tamarense*. Neither of the two *sxt* fragments were amplified from two *A. andersoni* and three *A. affine* strains. Homologs were also not detected in *Gambierdiscus australes*, *Amphidinium massartii*, *Prorocentrum lima*, *Ostreopsis siamensis* and *Ostreopsis ovata*, none of which are known to produce STX (Table 1).

Despite the very good correlation between the presence of *sxtA1* and *sxtA4* and STX content for most of the strains analysed, these fragments are not unambiguous markers for toxicity (Table 1). Both fragments were also amplified from *A. tamarense* strains for which no STX-production was detected (Table 1). Furthermore, RACE analyses of *A. tamarense* strain CCMP1771 revealed that *sxtA1* and *sxtA4* were transcribed in this supposedly non-STX-producing strain (data not shown).

Several scenarios may explain the discrepancy between the presence of *sxtA1*, *sxtA4* and toxin production: 1) other genes of the STX pathway are missing in these strains, 2) post-transcriptional mechanisms differ between STX-producing and non-producing strains, or 3) the amount of STX these strains produce is lower than the detection limit of the HPLC/MS toxin determination methods used. Scenarios 1) and 2) can only be investigated when all core genes of the STX pathway have been fully characterized in STX-producing species. Scenario 3) might be a possible explanation in some cases, since a very sensitive saxiphilin assay used to investigate *A. tamarense* strain ATBB01 found it to be toxic, whereas the HPLC methods used in the same study [64], as well as toxin assays in the present study did not detect STX in the same strain (Table 1).

Transcript abundance has been suggested to be positively related to the number of gene copies present in a dinoflagellate genome [28]. Hence, it is possible that strains with low levels of STX have fewer copies of the *sxt* genes compared to those with greater STX-production. If this holds true, then the presence of *sxtA1* and *sxtA4* would indicate toxicity and molecular methods could be developed to detect STX-producing cells in the environment.

### Evolution of STX-synthesis in eukaryotes and its role in the diversification of *Alexandrium*

The cyanobacterial *sxt* genes are highly conserved between cyanobacteria species and the gene cluster is thought to have arisen at least 2100 million years ago [12]. Our results show that dinoflagellate *sxtA* transcripts that are phylogenetically closely related to a clade of the cyanobacteria *sxtA* sequences and other bacterial putative toxin-related genes (Fig. 3 & Fig. 4) also have the same domain structure as cyanobacterial *sxtA* genes (Fig. 1). We propose that this striking similarity is most likely due to a horizontal gene transfer (HGT) event between ancestral STX-producing bacteria and dinoflagellates. Within dinoflagellates, STX are produced by species of the genera *Alexandrium* and *Pyrodinium*, which belong to the family Gonyaulacaceae within the order Gonyaulacales, as well as by one species of the genus *Gymnodinium*, which belongs to the family Gymnodiniaceae in the order Gymnodiniales. Thus, these toxins are produced by two genera within one family and by a single species from a distant dinoflagellate order. This distribution of STX-synthesis within the dinoflagellates as well as the close relationship between *Alexandrium* and *Gymnodinium catenatum sxtA* sequences (Fig. 3, 4; Supporting Information S1, S2), suggests that the bacteria-to-dinoflagellate HGT likely took place prior to the origin of the genera *Alexandrium* and *Pyrodinium*, and was followed by a dinoflagellate-to-dinoflagellate transfer into *G. catenatum*. The extent of eukaryote-to-eukaryote HGTs is often underestimated due to difficulties in detecting such events, however, recent work highlights the importance and prevalence of such gene transfers [65], [66].

We were not able to resolve the relationship among the dinoflagellate s*xtA* sequences in this study, as most the internal nodes were not statistically supported (Supporting Information S1, S2). Therefore, it was not possible to determine with certainty whether the evolution of the *sxtA* genes mirrors that of the genus *Alexandrium*, or to determine the origins of a putative HGT from *Alexandrium* into *G. catenatum*. However, the *sxtA1* and *sxtA4* gene copies from multiple strains of *G. catenatum*, *A. minutum*, and *A. catenella* tended to be clustered by species indicating that their history reflects the evolution of these species. The non-amplification of *sxtA1* and *sxtA4* from the non-STX-producing species *A. affine* and *A. andersoni* may indicate that the *sxtA* genes have either been lost from these lineages or have mutated so much, that the primers developed here were not able to amplify them.

Our two *Alexandrium* EST datasets contained transcripts, which encoded homologs to the majority of core *sxt* genes identified from cyanobacteria (Table 3). Even though the similarity to the cyanobacterial *sxt* genes was often significant, it was much less than observed for *sxtA*. The closest hits were to other bacterial or eukaryotic genes present in the database. This indicates that different genes in the *sxt* pathway may have separate origins in dinoflagellates. Further work is required to elucidate the complex origins of this gene cluster and will lead to further advances regarding the genomes and molecular biology of these ancient and important microorganisms.

## Supporting Information

Supporting Information S1 **S** ***xtA1*** **phylogenetic tree.** Maximum likelihood topology is shown. Numbers on nodes represent bootstrap values of maximum likelihood and Bayesian analyses, respectively. Sequences in **bold** are transcript-derived sequences; either generated using RACE or are contigs from 454 read assembly.
(PDF)

Supporting Information S2 **S** ***xtA4*** **phylogenetic tree.** Maximum likelihood topology is shown. Numbers on nodes represent bootstrap values of maximum likelihood and Bayesian analyses, respectively. Sequences in **bold** are transcript-derived sequences; either generated using RACE or are contigs from 454 read assembly.
(PDF)

Supporting Information S3 **S3a:** Results from the SignalP analyses (http://www.cbs.dtu.dk/services/SignalP/) of the long *sxtA* transcript. **S3b:** Results from the SignalP analyses (http://www.cbs.dtu.dk/services/SignalP/) of the short *sxtA* transcript. **S3c:** Below are the results from the prediction of transmembrane helices within the first 200 residues of the long *sxtA* transcript using the TMHMM server v2.0 (http://www.cbs.dtu.dk/services/TMHMM/) and Kyte Doolittle plots with a window size of 19 (values above the red line may indicate transmembrane helices). **S3d:** Below are the results from the prediction of transmembrane helices within the first 200 residues of the short *sxtA* transcript using the TMHMM server v2.0 (http://www.cbs.dtu.dk/services/TMHMM/) Kyte Doolittle plots with a window size of 19 (values above the red line may indicate transmembrane helices).
(PDF)

## References

[1] Hallegraeff GM, Hallegraeff GM, Anderson DM, Cembella AD (1995). Harmful algal blooms: a global overview.. Manual of harmful marine marine microalgae. International Oceanographic
Commission (IOC) Manual and Guides.

[2] Hoagland P, Scatasta S, Granéli E, Turner T (2006). The economic effects of harmful algal blooms.. Ecology of Harmful Algae.

[3] Alam M, Ikawa M, Sasner J, Sawyer P (1973). Purification of *Aphanizomenon flos-aquae* toxin
and its chemical and physiological properties.. Toxicon.

[4] Schantz EJ, Lynch JM, Vayvada G, Matsumot K, Rapoport H (1966). Purification and characterization of poison produced by
*Gonyaulax catenella* in axenic culture.. Biochemistry.

[5] Shimizu Y (1996). Microalgal metabolites: a new perspective.. Annu Rev Microbiol.

[6] Kellmann R, Mihali TK, Jeon YJ, Pickford R, Pomati F (2008). Biosynthetic intermediate analysis and functional homology reveal
a saxitoxin gene cluster in cyanobacteria.. Appl Environ Microbiol.

[7] Mihali TK, Kellmann R, Neilan BA (2009). Characterisation of the paralytic shellfish toxin biosynthesis
gene clusters in *Anabaena circinalis* AWQC131C and
*Aphanizomenon* sp. NH-5.. BMC Biochem.

[8] Moustafa A, Loram JE, Hackett JD, Anderson DM, Plumley FG (2009). Origin of saxitoxin biosynthetic genes in
cyanobacteria.. PLoS ONE.

[9] Stucken K, John U, Cembella A, Murillo AA, Soto-Liebe K (2010). The smallest known genomes of multicellular and toxic
cyanobacteria: comparison, minimal gene sets for linked traits and the
evolutionary implications.. PLoS ONE.

[10] Mihali TK, Carmichael WW, Neilan BA (2011). A putative gene cluster from a *Lyngbya wollei*
bloom that encodes paralytic shellfish toxin biosynthesis.. PloS ONE.

[11] Wiese M, D'Agostino PM, Mihali TK, Moffitt MC, Neilan BA (2010). Neurotoxic alkaloids: saxitoxin and its analogs.. Marine Drugs.

[12] Murray SA, Mihali TK, Neilan BA (2011). Extraordinary conservation, gene loss and positive selection in
the evolution of an ancient neurotoxin.. Mol Biol Evol.

[13] Sako Y, Yoshida T, Uchida A, Arakawa O, Noguchi T (2001). Purification and characterization of a sulfotransferase specific
to N-21 of saxitoxin and gonyautoxin 2+3 from the toxic dinoflagellate
*Gymnodinium catenatum* (Dinophyceae).. J Phycol.

[14] Yoshida T, Sako Y, Uchida A, Kakutani T, Arakawa O (2002). Purification and characterization of sulfotransferase specific to
O-22 of 11-hydroxy saxitoxin from the toxic dinoflagellate
*Gymnodinium catenatum* (dinophyceae).. Fisheries Science.

[15] Taroncher-Oldenburg G, Kulis DM, Anderson DM (1999). Coupling of saxitoxin biosynthesis to the G1 phase of the cell
cycle in the dinoflagellate *Alexandrin fundyense*:
temperature and nutrient effects.. Nat Toxins.

[16] Taroncher-Oldenburg G, Anderson DM (2000). Identification and characterization of three differentially
expressed genes, encoding S-adenosylhomocysteine hydrolase, methionine
aminopeptidase, and a histone-like protein, in the toxic dinoflagellate
*Alexandrium fundyense*.. Appl Environ Microbiol.

[17] Plumley FG (2001). Purification of an enzyme involved in saxitoxin
synthesis.. J Phycol.

[18] Harlow LD, Koutoulis A, Hallegraeff GM (2007). S-adenosylmethionine synthetase genes from eleven marine
dinoflagellates.. Phycologia.

[19] Yang I, John U, Beszteri S, Glöckner G, Krock B (2010). Comparative gene expression in toxic versus non-toxic strains of
the marine dinoflagellate *Alexandrium
minutum*.. BMC Genomics.

[20] Kellmann R, Stüken A, Orr RJS, Svendsen HM, Jakobsen KS (2010). Biosynthesis and molecular genetics of polyketides in marine
dinoflagellates.. Marine Drugs.

[21] Piel J (2004). Metabolites from symbiotic bacteria.. Nat Prod Rep.

[22] Hold GL, Smith EA, Birkbeck TH, Gallacher S (2001). Comparison of paralytic shellfish toxin (PST) production by the
dinoflagellates *Alexandrium lusitanicum* NEPCC 253 and
*Alexandrium tamarense* NEPCC 407 in the presence and
absence of bacteria.. FEMS Microbiol Ecol.

[23] Sato S, Shimizu Y (1998). Purification of a fluorescent product from the bacterium
*Moraxella*: a neosaxitoxin imposter.

[24] Prol MJ, Guisande C, Barreiro A, Míguez B, de la Iglesia P (2009). Evaluation of the production of paralytic shellfish poisoning
toxins by extracellular bacteria isolated from the toxic dinoflagellate
*Alexandrium minutum*.. Can J Microbiol.

[25] Baker TR, Doucette GJ, Powell CL, Boyer GL, Plumley FG (2003). GTX(4) imposters: characterization of fluorescent compounds
synthesized by *Pseudomonas stutzeri* SF/PS and
*Pseudomonas*/*Alteromonas* PTB-1,
symbionts of saxitoxin-producing *Alexandrium*
spp.. Toxicon.

[26] Martins CA, Alvito P, Tavares MJ, Pereira P, Doucette G (2003). Reevaluation of production of paralytic shellfish toxin by
bacteria associated with dinoflagellates of the Portuguese
coast.. Appl Environ Microbiol.

[27] Le QH, Markovic P, Hastings J, Jovine RVM, Morse D (1997). Structure and organization of the peridinin-chlorophyll a-binding
protein gene in *Gonyaulax polyedra*.. Mol Gen Genet.

[28] Bachvaroff TR, Place AR (2008). From stop to start: tandem gene arrangement, copy number and
trans-splicing sites in the dinoflagellate *Amphidinium
carterae*.. PLoS ONE.

[29] Zhang H, Hou Y, Lin S (2006). Isolation and characterization of proliferating cell nuclear
antigen from the dinoflagellate *Pfiesteria
piscicida*.. J Eukaryot Microbiol.

[30] Moustafa A, Evans AN, Kulis DM, Hackett JD, Erdner DL (2010). Transcriptome profiling of a toxic dinoflagellate reveals a
gene-rich protist and a potential impact on gene expression due to bacterial
presence.. PLoS ONE.

[31] Zhang H, Hou Y, Miranda L, Campbell DA, Sturm NR (2007). Spliced leader RNA trans-splicing in
dinoflagellates.. Proc Natl Acad Sci USA.

[32] Lidie KB, van Dolah FM (2007). Spliced leader RNA-mediated trans-splicing in a dinoflagellate,
*Karenia brevis*.. J Eukaryot Microbiol.

[33] Hastings KEM (2005). SL trans-splicing: easy come or easy go?. Trends Genet.

[34] Cavaliere R (2008). The regulation of saxitoxin production in cyanobacteria. PhD
thesis.

[35] Doblin MA, Blackburn SI, Hallegraeff GM (1999). Growth and biomass stimulation of the toxic dinoflagellate
*Gymnodinium catenatum* (Graham) by dissolved organic
substances.. J Exp Mar Biol Ecol.

[36] Guillard RRL, Hargraves PE (1993). *Stichochrysis immobilis* is a diatom, not a
chyrsophyte.. Phycologia.

[37] Orr RJS, Stüken A, Rundberget T, Eikrem W, Jakobsen KS (under revision). Improved phylogenetic resolution of toxic and non-toxic
*Alexandrium* strains using a concatenated rDNA
approach.. Harmful Algae.

[38] Jørgensen MF, Murray S, Daugbjerg N (2004). *Amphidinium* revisited. I. Redefinition of
*Amphidinium* (Dinophyceae) based on cladistic and
molecular phylogenetic analyses.. J Phycol.

[39] Kumar S, Carlsen T, Mevik B-H, Enger P, Blaalid R (in revision). CLOTU: An online pipeline for processing and clustering of 454
amplicon reads into OTUs followed by taxonomic annotation.. Bioinformatics.

[40] Chevreux B, Pfisterer T, Drescher B, Driesel AJ, Muller WEG (2004). Using the miraEST assembler for reliable and automated mRNA
transcript assembly and SNP detection in sequenced ESTs.. Genome Res.

[41] Kellmann R (2005). The molecular genetics of cylindrospermopsin and saxitoxin biosynthesis.
PhD thesis.

[42] Bendtsen JD, Nielsen H, von Heijne G, Brunak S (2004). Improved prediction of signal peptides: SignalP
3.0.. J Mol Biol.

[43] Shen HB, Chou KC (2007). Signal-3L: A 3-layer approach for predicting signal
peptides.. Biochem Biophys Res Commun.

[44] Kyte J, Doolittle RF (1982). A simple method for displaying the hydropathic character of a
protein.. J Mol Biol.

[45] Maddison W, Maddison D (1992). MacClade..

[46] Katoh K, Toh H (2008). Recent developments in the MAFFT multiple sequence alignment
program.. Brief Bioinform.

[47] Abascal F, Zardoya R, Posada D (2005). ProtTest: selection of best-fit models of protein
evolution.. Bioinformatics.

[48] Stamatakis A (2006). RAxML-VI-HPC: Maximum likelihood-based phylogenetic analyses with
thousands of taxa and mixed models.. Bioinformatics.

[49] Lartillot N, Philippe H (2004). A Bayesian mixture model for across-site heterogeneities in the
amino-acid replacement process.. Mol Biol Evol.

[50] Lartillot N, Philippe H (2006). Computing Bayes factors using thermodynamic
integration.. Syst Biol.

[51] Patron N, Waller R, Archibald J, Keeling P (2005). Complex protein targeting to dinoflagellate
plastids.. J Mol Biol.

[52] Lin S (2006). The smallest dinoflagellate genome is yet to be found: A comment
on LaJeunesse et al. “*Symbiodinium* (Pyrrhophyta)
genome sizes (DNA content) are smallest among
dinoflagellates”.. J Phycol.

[53] Hou Y, Lin S (2009). Distinct gene number-genome size relationships for eukaryotes and
non-eukaryotes: gene content estimation for dinoflagellate
genomes.. PLoS ONE.

[54] Stein LD (2004). Human genome: end of the beginning.. Nature.

[55] Ho AYT, Hsieh DPH, Qian PY (2006). Variations in paralytic shellfish toxin and homolog production in
two strains of *Alexandrium tamarense* after antibiotic
treatments.. Aquat Microb Ecol.

[56] Hackett JD, Scheetz TE, Yoon HS, Soares MB, Bonaldo MF (2005). Insights into a dinoflagellate genome through expressed sequence
tag analysis.. BMC Genomics.

[57] Erdner DL, Anderson DM (2006). Global transcriptional profiling of the toxic dinoflagellate
*Alexandrium fundyense* using massively parallel
signature sequencing.. BMC Genomics.

[58] Uribe P, Fuentes D, Valdes J, Shmaryahu A, Zuniga A (2008). Preparation and analysis of an Expressed Sequence Tag library
from the toxic dinoflagellate *Alexandrium
catenella*.. Mar Biotechnol.

[59] Hsiao YY, Lin CH, Liu JK, Wong TY, Kuo J (2010). Analysis of codon usage patterns in toxic dinoflagellate
*Alexandrium tamarense* through Expressed Sequence Tag
data.. Comp Funct Genom.

[60] Stüken A, Jakobsen KS (2010). The cylindrospermopsin gene cluster of
*Aphanizomenon* sp strain 10E6: organization and
recombination.. Microbiology-Sgm.

[61] JCVI (2010). J Craig Venter Institute.

[62] Slamovits CH, Keeling PJ (2008). Widespread recycling of processed cDNAs in
dinoflagellates.. Current Biology.

[63] Zhang H, Dungan CF, Lin S (2010). Introns, alternative splicing, spliced leader trans-splicing and
differential expression of pcna and cyclin in *Perkinsus
marinus*.. Protist.

[64] Negri A, Llewellyn L, Doyle J, Webster N, Frampton D (2003). Paralytic shellfish toxins are restricted to few species among
Australia's taxonomic diversity of cultured microalgae.. J Phycol.

[65] Keeling PJ, Palmer JD (2008). Horizontal gene transfer in eukaryotic evolution.. Nat Rev Genet.

[66] Worden AZ, Allen AE (2010). The voyage of the microbial eukaryote.. Curr Opin Microbiol.

