# Supplementary figures and images for: Discovery of Nuclear-Encoded Genes for the Neurotoxin Saxitoxin in Dinoflagellates
Source: PLoS One. 2011 May 18;6(5):e20096. doi: 10.1371/journal.pone.0020096 (PMC3097229; doi:10.1371/journal.pone.0020096)

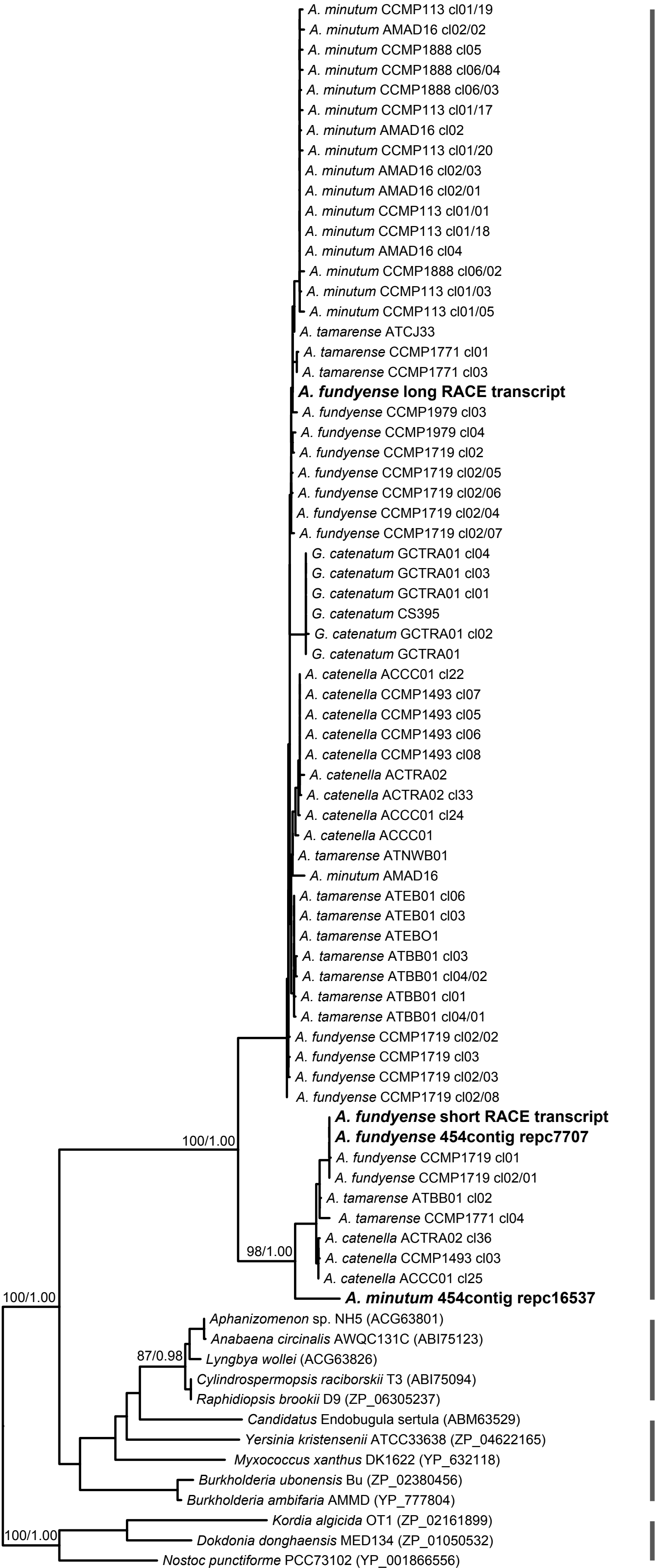

Dinoflagellate  
*sxtA1*

Cyanobacteria  
*sxtA1*

Proteobacteria

OUTGROUP

Supplement: Supporting Information S1 — S xtA1 phylogenetic tree. Maximum likelihood topology is shown. Numbers on nodes represent bootstrap values of maximum likelihood and Bayesian analyses, respectively. Sequences in bold are transcript-derived sequences; either generated using RACE or are contigs from 454 read assembly. (PDF) [file pone.0020096.s001.pdf]

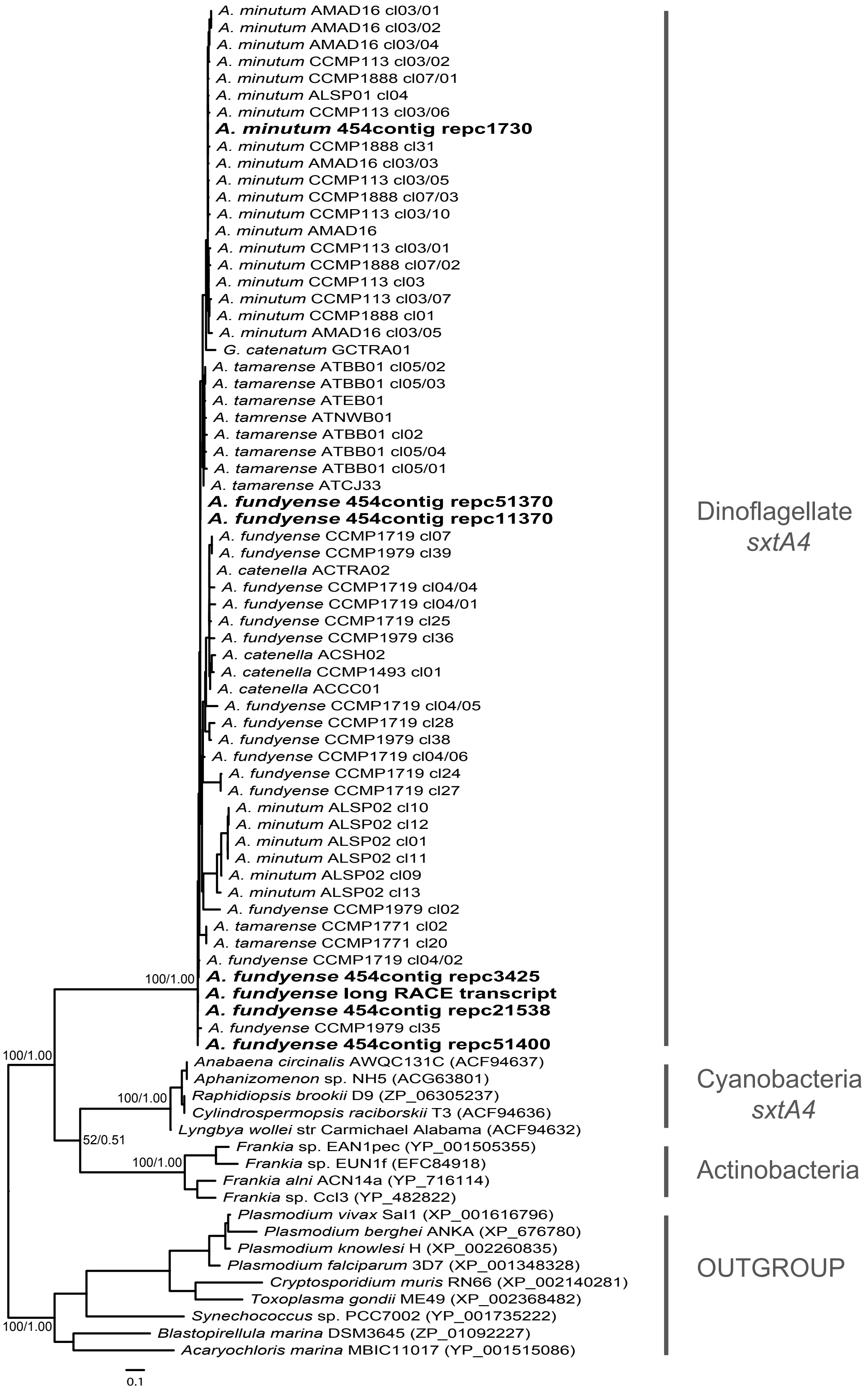

Supplement: Supporting Information S2 — S xtA4 phylogenetic tree. Maximum likelihood topology is shown. Numbers on nodes represent bootstrap values of maximum likelihood and Bayesian analyses, respectively. Sequences in bold are transcript-derived sequences; either generated using RACE or are contigs from 454 read assembly. (PDF) [file pone.0020096.s002.pdf]
