# Supplementary material for: Discovery of Nuclear-Encoded Genes for the Neurotoxin Saxitoxin in Dinoflagellates
Source: PLoS One. 2011 May 18;6(5):e20096. doi: 10.1371/journal.pone.0020096 (PMC3097229; doi:10.1371/journal.pone.0020096)

Supporting Information S3a:

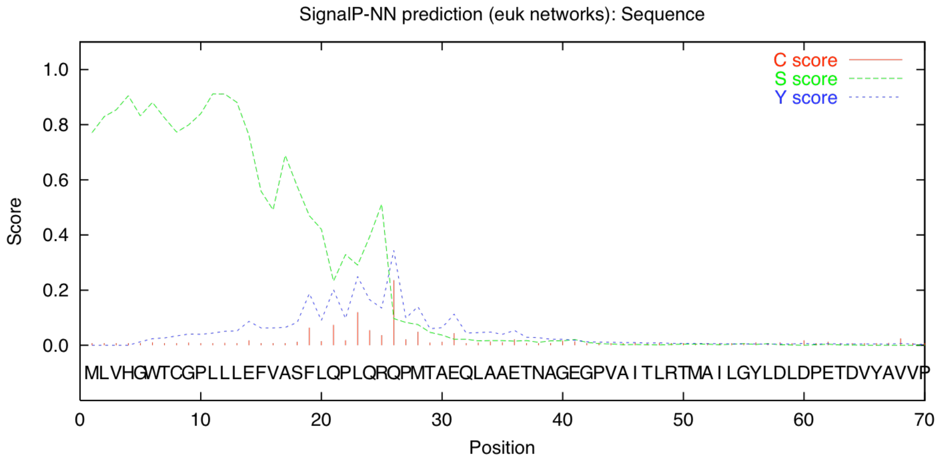


```
>Sequence                length = 70
# Measure       Position  Value  Cutoff  signal peptide?
max. C          26         0.236  0.32    NO
max. Y          26         0.344  0.33    YES
max. S          11         0.912  0.87    YES
mean S          1-25       0.670  0.48    YES
D               1-25       0.507  0.43    YES
# Most likely cleavage site between pos. 25 and 26: LQR-QP
```

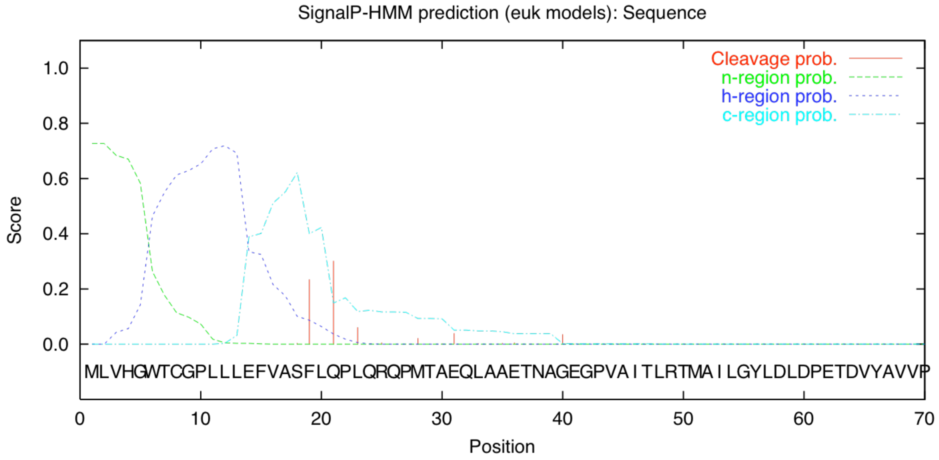


```
>Sequence Prediction: Signal peptide
Signal peptide probability: 0.727
Signal anchor probability: 0.000
Max cleavage site probability: 0.302 between pos. 20 and 21
```

Supporting Information S3b:

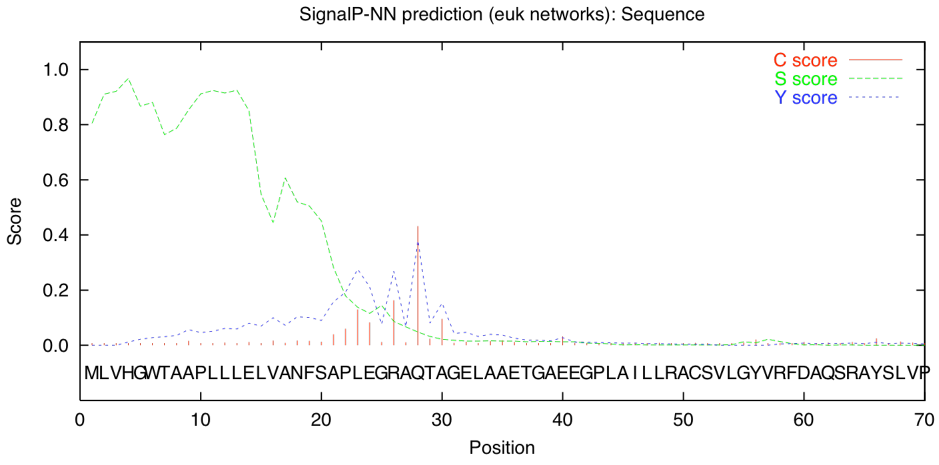

>Sequence length = 70

# Measure	Position	Value	Cutoff	signal peptide?
max. C	28	0.432	0.32	YES
max. Y	28	0.380	0.33	YES
max. S	4	0.968	0.87	YES
mean S	1-27	0.606	0.48	YES
D	1-27	0.493	0.43	YES

Most likely cleavage site between pos. 27 and 28: GRA-QT

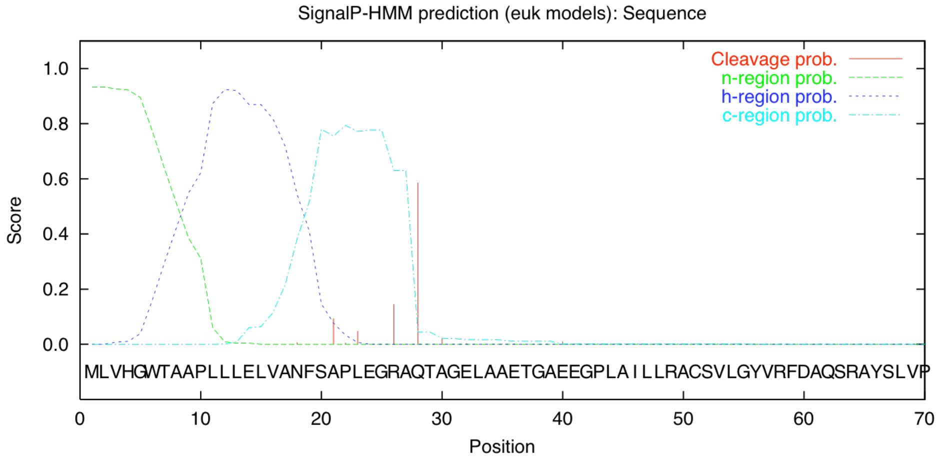

>Sequence Prediction: Signal peptide
Signal peptide probability: 0.933
Signal anchor probability: 0.000
Max cleavage site probability: 0.587 between pos. 27 and 28

Supporting Information S3c:

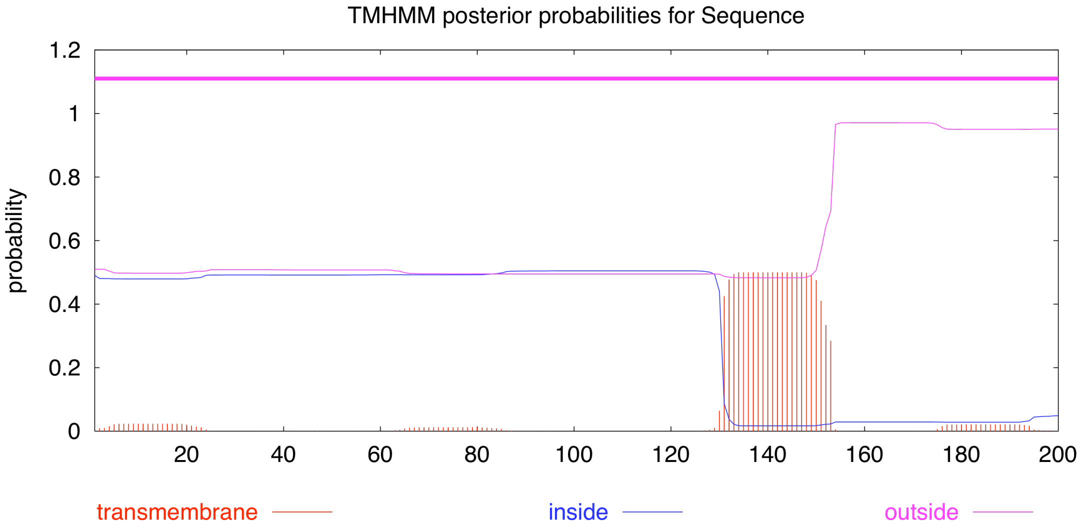

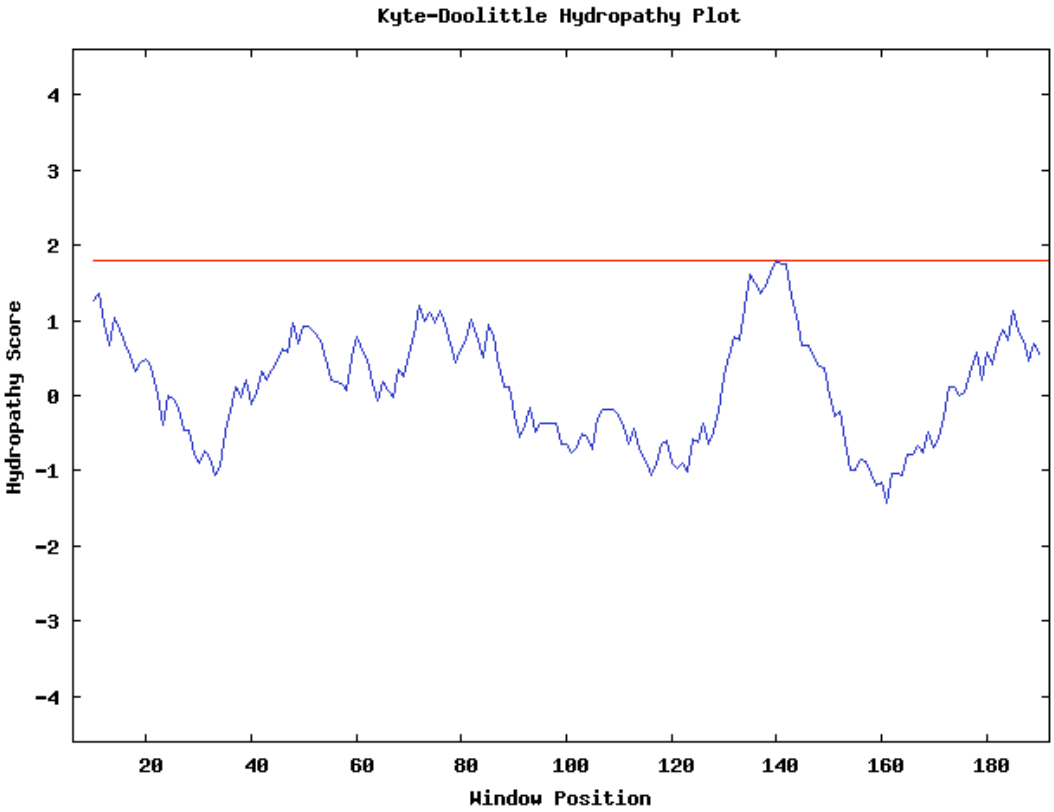

Supporting Information S3d:

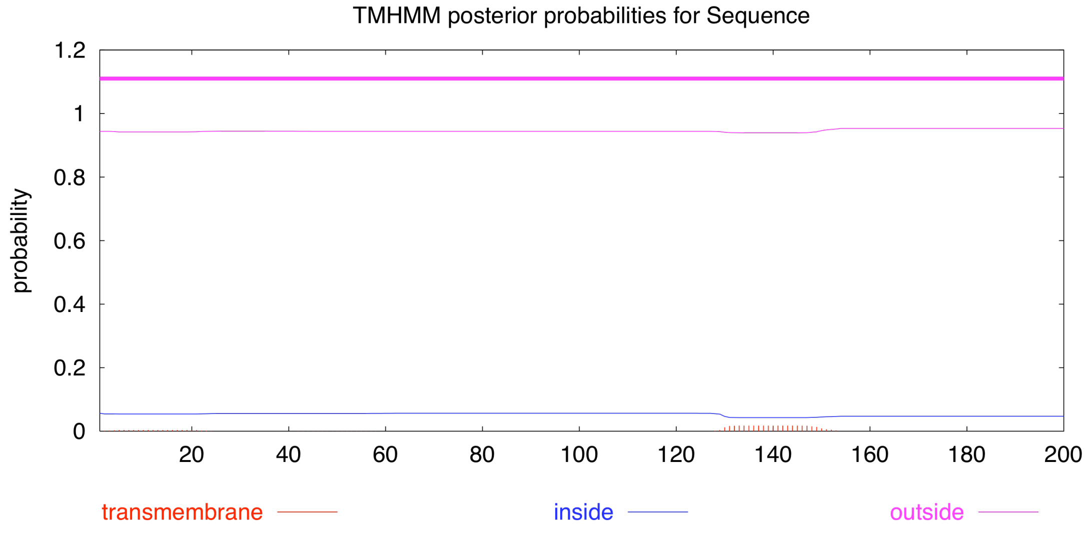

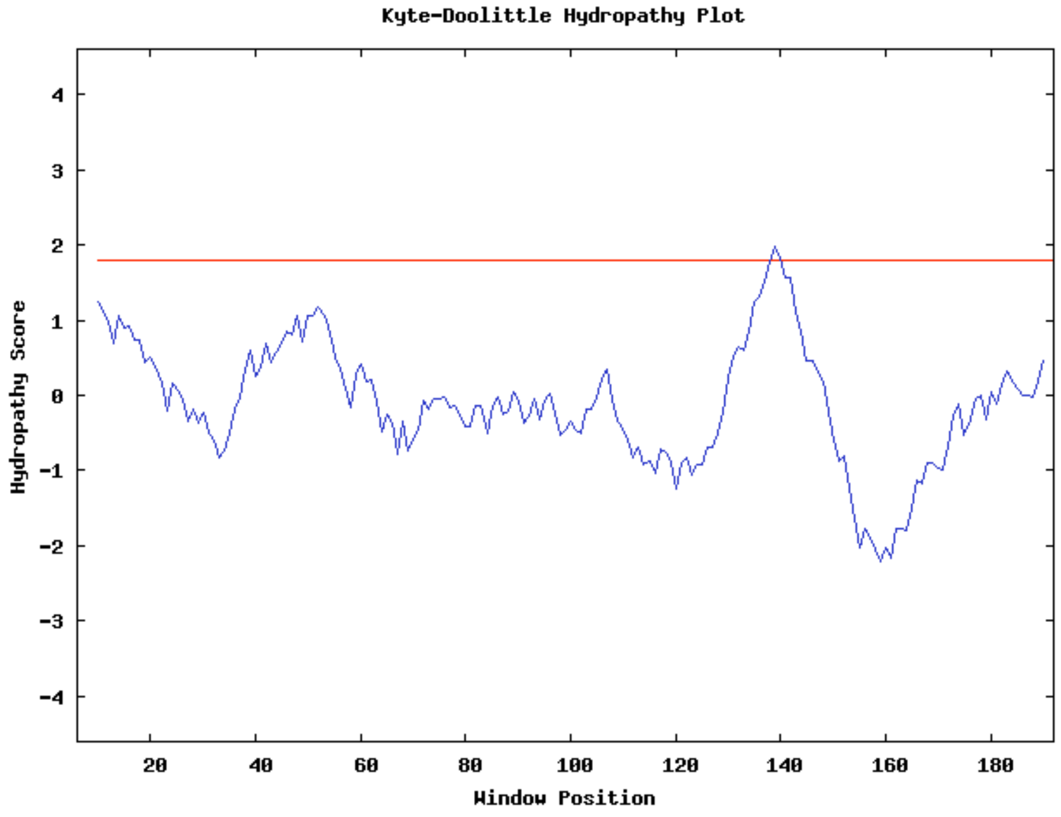

Supplement: Supporting Information S3 — S3a: Results from the SignalP analyses (http://www.cbs.dtu.dk/services/SignalP/) of the long sxtA transcript. S3b: Results from the SignalP analyses (http://www.cbs.dtu.dk/services/SignalP/) of the short sxtA transcript. S3c: Below are the results from the prediction of transmembrane helices within the first 200 residues of the long sxtA transcript using the TMHMM server v2.0 (http://www.cbs.dtu.dk/services/TMHMM/) and Kyte Doolittle plots with a window size of 19 (values above the red line may indicate transmembrane helices). S3d: Below are the results from the prediction of transmembrane helices within the first 200 residues of the short sxtA transcript using the TMHMM server v2.0 (http://www.cbs.dtu.dk/services/TMHMM/) Kyte Doolittle plots with a window size of 19 (values above the red line may indicate transmembrane helices). (PDF) [file pone.0020096.s003.pdf]
